# Impact study of data locality on task-based applications through the Heteroprio scheduler

**DOI:** 10.7717/peerj-cs.190

**Published:** 2019-05-06

**Authors:** Bérenger Bramas

**Affiliations:** CAMUS Team, Inria Nancy—Grand Est, Illkirch-Graffenstaden, France

**Keywords:** Scheduling, Task-based, Starpu, HPC, Data locality

## Abstract

The task-based approach has emerged as a viable way to effectively use modern heterogeneous computing nodes. It allows the development of parallel applications with an abstraction of the hardware by delegating task distribution and load balancing to a dynamic scheduler. In this organization, the scheduler is the most critical component that solves the DAG scheduling problem in order to select the right processing unit for the computation of each task. In this work, we extend our Heteroprio scheduler that was originally created to execute the fast multipole method on multi-GPUs nodes. We improve Heteroprio by taking into account data locality during task distribution. The main principle is to use different task-lists for the different memory nodes and to investigate how locality affinity between the tasks and the different memory nodes can be evaluated without looking at the tasks’ dependencies. We evaluate the benefit of our method on two linear algebra applications and a stencil code. We show that simple heuristics can provide significant performance improvement and cut by more than half the total memory transfer of an execution.

## Introduction

High-performance computing (HPC) is crucial to make advances and discoveries in numerous domains. However, while supercomputers are becoming more powerful, their complexity and heterogeneity also increase; in 2018, a quarter of the most powerful supercomputers in the world are equipped with accelerators (see https://www.top500.org/), and the majority of them (including the top two on the list) uses GPUs in addition to traditional multi-core CPUs. The efficient use of these machines and their programmability are ongoing research topics. The objectives are to allow the development of efficient computational kernels for the different processing units and to create the mechanisms to balance the workload and copy/distribute the data between the CPUs and the devices. Furthermore, this complexity forces some of the scientific computing developers to alternate computation on CPUs or GPUs, but never use both at the same time. This naive parallelization scheme usually provides a speedup compared to a CPU-only execution, but it ends in wastage of computational resources and utilization of extra barrier synchronizations.

Meanwhile, the HPC community has proposed several strategies to parallelize applications on heterogeneous computing nodes with the aim of using all available resources. Among the existing methods, the task-based approach has gained popularity: mainly because it makes it possible to create parallel codes with an abstraction of the hardware by delegating the task distribution and load balancing to dynamic schedulers. In this method, the workload is split into inter-dependent computational elements and is managed by a runtime system (RS). There are several RS reported in the literature (Danalis et al., 2014; Kale & Krishnan, 1993; Perez, Badia & Labarta, 2008; Gautier et al., 2013; Bauer et al., 2012; Tillenius, 2015), and each of them has its own specificity and interface. We refer to a comparative study (Thoman et al., 2018) for a detailed description where the different aspects and features of RS are categorized. Task-based method is a viable solution to use modern heterogeneous computing nodes and mix computation between CPU and devices. Furthermore, the potential of this approach has already been proven on numerous computational methods. In the task-based method, the scheduler is in charge of the most important decisions, as it has to decide the order of computation of the ready tasks (the tasks that have their dependencies satisfied) as well as where those tasks should be computed. In the present study, we implemented our scheduler inside a RSs called StarPU (Augonnet et al., 2011), which supports heterogeneous architectures and allows customizing the scheduler in an elegant manner.

In our previous work, we created the Heteroprio scheduler to execute the fast multipole method (FMM) using StarPU on computing nodes equipped with multiple GPUs (Agullo et al., 2016b). Heteroprio was first implemented inside ScalFMM (Bramas, 2016), and it was later included in StarPU. It is publicly available and usable by any StarPU-based code. In fact, Heteroprio was later used in linear algebra applications where it demonstrated its robustness and potential, see QrMUMPS (Agullo et al., 2015) and SpLDLT (Lopez & Duff, 2018). Moreover, it was also the subject of theoretical studies (Beaumont et al., 2016; Beaumont, Eyraud-Dubois & Kumar, 2017, 2018; Agullo et al., 2016a), which revealed its advantages and gave a positive theoretical insight on the performance. However, the original Heteroprio scheduler does not take into account data locality. The distribution of the tasks—the choice of the processing unit that will compute a given task—is done without considering the distribution of the data. Therefore, depending on the applications and the test cases, Heteroprio can not only lead to huge data movement between CPUs and GPUs but also between GPUs, which dramatically penalizes the execution. The current work proposed different mechanisms to consider data locality in order to reduce the data transfers and the makespan.

The contributions of this paper are as follows:

- We summarize the main ideas of the Heteroprio scheduler and explain how it can be implemented in a simple and efficient manner;
- We propose new mechanisms to include data locality in the Heteroprio scheduler’s decision model;
- We define different formulas to express the locality affinity for a given task relative to the different memory nodes. Those formulas are based on general information regarding the hardware or the data accesses;
- We evaluate our approach on two linear algebra applications, QrMumps and SpLDLT, and a stencil application, and analyze the effect of the different parameters.

The rest of the paper is organized as follows. In the section “Background,” we introduce the task-based parallelization and the original Heteroprio scheduler. Then, in the section “Introducing Laheteroprio,” we detail our new methods to use data locality and the different mechanisms of our locality-aware Heteroprio (LAHeteroprio) scheduler. Finally, we evaluate our approach in the section “Performance Study” by plugging in the LAHeteroprio inside StarPU to execute two different linear algebra applications using up to four GPUs.

## Background

### Task-based parallelization

The task-based approach divides an application into interdependent sections, called tasks, and provides the dependencies between them. These dependencies allow valid parallel executions, that is, with a correct execution order of the tasks and without race conditions. This description can be viewed as a graph where the nodes represent the tasks and the edges represent the dependencies. If the edges represent a relation of precedence between the tasks, the resulting graph is a direct acyclic graph of tasks. However, this is not the case when an inter-tasks dependency relation is used, such as a mechanism to express that an operation is commutative (Agullo et al., 2017). In the paper, we consider graphs of the form *G* = (*V*, *E*) with a set of nodes *V* and a set of edges *E*. Considering *t*_1_,*t*_2_ ∈ *V*, there exists a relation (*t*_1_,*t*_2_) ∈ *E*—also written *t*_1_ → *t*_2_—if the task *t*_2_ can be executed only after the task *t*_1_ is over.

A task *t* is a computational element that is executable on one or (potentially) several different types of hardware. When *t* is created, it incorporates different interchangeable kernels where each of them targets a different architecture. For example, consider a matrix-matrix multiplication task in linear algebra: it could be either a call to cuBLAS and executed on a GPU, or a call to Intel MKL and executed on a CPU, but both kernels return a result that is considered equivalent. Task *t* accesses data either in *read*, *read-write* or *write* and in the rest of the paper we consider equivalent the *read-write* and the *write* accesses. We denote *t.data* to be the set of data elements that *t* will access during its execution. From this information, that is, *G* = (*V*, *E*) and the portability of the tasks, the scheduler must decide the order of computation and where to execute the tasks.

### Task scheduling and related work

Scheduling can be done statically or dynamically, and in both cases, finding an optimal distribution of the tasks is usually NP complete since the solution must find the best computing order and the best processing unit for each task (Peter Brucker, 2009).

The static approaches have a view on the complete set of tasks before the beginning of the execution (Baptiste, Pape & Nuijten, 2001), and thus can use expensive mechanisms to analyze the relationship between the tasks. Advanced strategies are also used, such as duplicating tasks to replace communications with computation (He et al., 2019). It is worth mentioning that these strategies can have significant overhead compared to their benefit and the execution time of the tasks, which make them unusable in real applications. Static scheduling requires performance models, so it can predict the duration of the tasks on the different architectures and the duration of the communications. Even, if it is possible to build such systems, they require costly calibration/evaluation stages and their resulting prediction models are not always accurate, especially in the case of irregular applications. Moreover, these approaches cannot adapt their executions to the unpredictable noise generated by the OS or the hardware.

**Figure 1:**
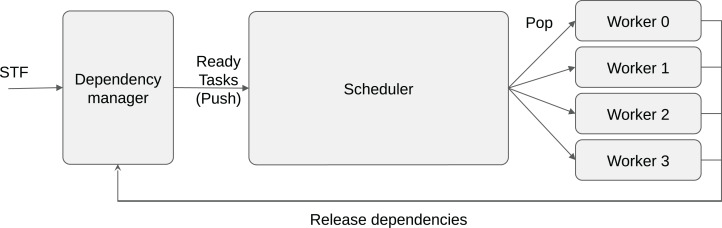
Schematic view of task-based runtime system organization. A program can be described using the sequential task flow (STF) model and converted into tasks/dependencies by the RS. When dependencies are released, the newly ready tasks are pushed into the scheduler. When a worker is idle, it calls the pop function of the scheduler to request a task to execute.

This is why most task-based applications use RSs that are powered with dynamic scheduling strategies (Akbudak et al., 2018; Sukkari et al., 2018; Moustafa et al., 2018; Carpaye, Roman & Brenner, 2018; Agullo et al., 2016b). In this case, the scheduler focuses only on the ready tasks and decides during the execution on how to distribute them. It has been demonstrated that these strategies are able to deliver high performance with reduced overhead. The scheduler becomes a critical layer of the RS, at the boundary between the dependencies manager and the workers, see Fig. 1. We follow the StarPU’s terminology and consider that a scheduler has an entry point where the ready tasks are pushed, and it provides a request method where workers pop the tasks to execute. In StarPU, both pop/push methods are directly called by the workers that either release the dependencies or ask for a task. Consequently, assigning a task to a given worker means to return this task when the worker calls the pop method.

As an intuitive example, consider a priority-based scheduler designed to manage priorities with one task-list per priority. The push method can simply store a newly ready task *t* in the right list *list[t.priority].push_back(t)*. Meanwhile, the pop method can iterate over the lists and when it finds one non-empty list, it pops a task from it. Furthermore, in the case of heterogeneous computing, a pop must return a task compatible with the worker that performs the request.

Managing data locality was already a challenge before the use of heterogeneous computing because of NUMA hardware and a simple scheduling strategy has been proposed to improve data locality on the NUMA nodes (Al-Omairy et al., 2015). Past work has introduced distance-aware work-stealing scheduling heuristics within the OmpSs runtime, targeting dense linear algebra applications on homogeneous *x*86 hardware. While the method provides a significant speedup, it does not take into account the different data accesses (read or write) or look at the cache levels to find data replication.

The importance of data locality to move forward with exascale computing has been emphasized (Unat et al., 2017) with a focus on task-based RSs. The authors shown that data movement is now the primary source of energy consumption in HPC.

In era of heterogeneous computing, the community has provided various strategies to schedule graphs of tasks on this kind of architecture, and one of the most famous is the heterogeneous earliest finish time (HEFT) scheduler (Topcuoglu, Hariri & Wu, 2002). In HEFT, tasks are prioritized based on a heuristic that takes into account a prediction of the duration of the tasks and the data transfers between tasks. Different models exist, but on a heterogeneous computing node, the duration of a task can be the average duration of the task on the different types of processing unit. More advanced ranking models had been defined (Shetti, Fahmy & Bretschneider, 2013). However, this scheduler has two limitations that we would like to alleviate. First, it uses a prediction system, which may need an important tuning stage and may be inaccurate, as we previously argued. Second, even if ranking a set of tasks can be amortized and beneficial, re-ranking the tasks to consider new information concerning the ongoing execution can add a dramatic overhead. This is why we have proposed an alternative scheduler.

### Heteroprio

#### Multi-priorities

Within Heteroprio, we assign one priority per processing unit type to each task, such that a task has several priorities. Each worker pops the task that has the highest priority for the hardware type it uses, which are CPU or GPU in the present study. With this mechanism, each type of processing unit has its own priority space. This allows to continue using priorities to manage the critical path, and also to promote the consumption of tasks by the more *appropriate* workers: workers do first what they are good at.

**Figure 2:**
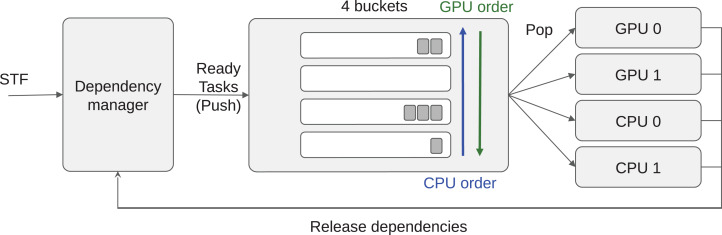
Heteroprio schematic view. Tasks are pushed inside the buckets. Each worker iterates on the buckets based on the priorities for the hardware it uses.

The tasks are stored inside buckets, where each bucket corresponds to a priority set. Then each worker uses an indirect access array to know the order in which it should access the buckets. Moreover, all the tasks inside a bucket must be compatible with all the processing units that may access it (at least). This allows an efficient implementation. As a result, we have a constant complexity for the push and complexity of *O*(*B*) for the pop, where *B* is the number of buckets. The number of buckets *B* corresponds to the number of priority groups, which is equal to the number of different operation types in most cases. A schematic view of the scheduler is provided in Fig. 2.

For illustration, let us consider an application with four different types of task *T_A_*, *T_B_*, *T_C_* and *T_C_*_′_ (here *T_C_* and *T_C_*_′_ can be the same operation but with data of small or large granularity, respectively). Tasks of types *T_A_*, *T_C_* and *T*_*C*′_ provide a kernel for CPU and GPU and thus are executable on both, but tasks of type *T_B_* are only compatible with CPUs. Consequently, we know that GPU workers do not access the bucket where *T_B_* tasks are stored. Then, we consider that the priorities on CPU are *P*_CPU_(*T_A_*) = 0, *P*_CPU_(*T_B_*) = 1, *P*_CPU_(*T_C_*) = 2 and *P*_CPU_(*T_C_*_′_) = 3; on GPU the priorities are *P*_GPU_(*T_A_*) = 1, *P*_GPU_(*T_C_*) = 0 and *P*_GPU_(*T_C_*_′_) = 0. We highlight that *T_C_* and *T_C_*_′_ have the same priority for GPU workers. From this configuration, we end with four buckets: *B*_0_ = {*T_A_*}, *B*_1_ = {*T_B_*}, *B*_2_ = {*T_C_*} and *B*_3_ = {*T_C_*_′_}. Finally, the indirect access arrays are *A*_CPU_ = {0,1,2,3} and *A*_GPU_ = {3,2,0} with *A*_GPU_ = {2,3,0} being valid as well.

#### Speedup factors

The *speedup factors* are used to manage the critical moments when a low number of ready tasks are available. The idea is to forbid some workers to get a task from a set of buckets when their corresponding hardware type is not the fastest to compute the buckets’ tasks. To do so, the type of processing unit that is the fastest in average to execute the bucket’s tasks, is provided for each bucket. Additionally, we input a number that indicates by how much this processing unit type is faster compared to the other types of processing units. These numbers are used to define a limit under which the slow workers cannot pick a task.

As an illustration, let us consider two types of processing units: CPU and GPU. Let *S_i_* be the speedup factor for bucket *i* and let GPU be the fastest type to compute the task stored in *i*. A CPU worker can take a task from bucket *i* if there are more than *N*_GPU_ × *S_i_* available tasks in it, where *N*_GPU_ is the number of GPU workers. For example, if there are three GPU workers and that a GPU is two times faster in average than a CPU to perform a given operation, then a CPU worker takes a task only if there are six or more tasks available. Otherwise, it considers the bucket empty and continues to the next ones to find a task to compute. This means that for the example given in the section “Multi-Priorities,” we have two arrays of four items for the different operations, one to tell which processing units is the fastest, and a second one to provide the speedup. The description of the example tells us that the GPU cannot compute *T_A_*, so CPU are the fastest by default, and that *T_C_* and *T_C_*_′_ are the same operation but with different granularities, such that the speedup for the GPU will be higher for *T_C_*_′_ than *T_C_*. As a results, the arrays could be Best = {CPU, GPU, GPU, GPU} and Speedup = {1, 1.1, 1.4, 3}.

This system is used for each bucket individually and not globally. Therefore, if the number of buckets is large, this can lead to overflowing some workers and artificially keeping others idle. However, we found that in practice it provides beneficial results especially at the end of simulations.

## Introducing Laheteroprio

### 2D Task-list grid by splitting the buckets per memory nodes

**Figure 3:**
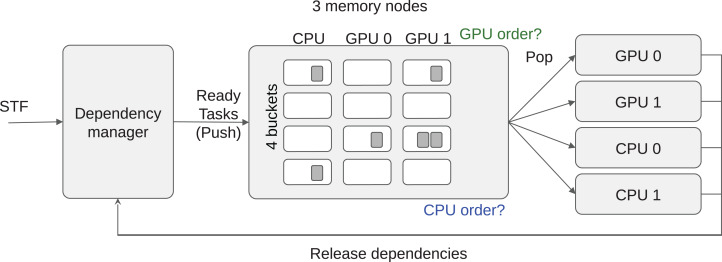
LAHeteroprio schematic view of a grid composed of four buckets and three memory nodes. The decision that the scheduler has to do is to put the tasks in the more appropriate lists and to decide how the workers iterate on the grid.

Our first step in managing data locality is to subdivide each bucket into *M* different task-lists and set up one list for each of the *M* memory nodes. For example, if the machine is composed of two GPUs and one CPU, we have three task-lists per bucket by considering NUMA memory nodes as a single one, without loss of generality. We obtain a 2D grid of task-lists *G* where the different buckets are in the first dimension and the memory nodes are in the second dimension, as illustrated in Fig. 3. We store in the list *G*(*b*,*m*) all the tasks of the bucket index *b* that we consider local to the memory node *m*. In this context, local means that an execution on a processing unit connected to *m* should have the lowest memory transfer cost. The list *G*(*b*,*m*) can also contain tasks that processing units connected to *m* cannot compute. This can happen when *m* is a GPU and the tasks of bucket index *b* do not provide a GPU function. Nevertheless, when workers steal tasks from *G*(*b*,*m*), we know that they have the highest affinity for the memory node *m* even if it is impossible to compute these tasks on a attached processing unit. From this description, we must provide a mechanism to find out the best memory node for every newly ready task, to push the tasks in the right list, and also decide how the workers should iterate on *G* and select a task.

Extending the example from the sections “Multi-Priorities” and “Speedup Factors,” the number of tasks list in each bucket is hardware specific because it corresponds to the number of memory nodes.

### Task insertion in the grid with locality evaluation (push)

In the original Heteroprio, there is no choice where a given task has to be stored, as it must be in the list of its corresponding bucket, that is, in *scheduler.list[task.bucket].push_back(task)*. On the other hand, in LAHeteroprio we have to decide in which list of the selected bucket we should put the task; we have to find the best *m* in *scheduler.list[task.bucket][m].push_back(task)*. Therefore, we propose different formulas to estimate the locality of a task regarding the memory nodes and the distribution of the data it uses.

The specificity of this approach is to determine the most suitable memory node without looking at the algorithm itself. We only look at each task individually without following the links it has with some other tasks and without making a prediction of how the pieces of data are going to move.

### Last recently used

In this strategy, we consider that the memory node related to the worker that pushes the task has the best locality; a newly ready task *t* released by worker *w* is pushed into *G*(*t.bucket*_*id*, *w.memory*_*node*). Indeed, *t* and the last task executed by *w* have at least one data dependency in common, and this data is already on the memory node if it has not been evicted. The main advantage of this technique is its simplicity and low overhead. However, it is obviously far from accurate. For example, it does not evaluate the amount of data that is already available on the memory node compared to the total amount of data that *t* will use.

Moving cost estimation seems natural to consider that the best memory node is the one that will allow moving the data in the shortest time. StarPU provides the function *starpu_task_expected _data_transfer_time_for* that predicts this transfer duration by looking where the pieces of data are and the possible transfer paths between the memory nodes. From this prediction, we obtain a moving cost and we refer to it as *MC_StarPU*.

### Data locality affinity formulas

StarPU’s prediction has two potential drawbacks: The first is that it treats all data dependencies similarly without making a distinction if the dependencies are *read* or *write*, and the second is that the memory transfer predictions are difficult to achieve since they are based on models that can be inaccurate and influenced by the on-going execution. Therefore, we propose different formulas to estimate the locality of a task and we obtain either a locality score for each memory node (the higher the better), or a moving cost (the lower the better). This information is used to decide where to put the newly ready tasks in the grid.

In our next formulas, we use the following notations

(1) $${D_{t,m}} = t.data \cap m.data, $$

(2) $${D_{t,\neg m}} = t.data \cap \neg m.data, $$

(3) $$D_{t,m}^{READ} = t.data \cap m.data\, \cap \,READ, $$

(4) $$D_{t,m}^{WRITE} = t.data \cap m.data \cap WRITE, $$

(5) $$READ \cap WRITE = \emptyset .$$

Here, *D_t_*_,*m*_ is the set of data used by task *t* and that exist on memory node *m*, whereas *D*_*t,¬m*_ represents the set of data used by *t* that is not on *m*. *D*^*READ*^_*t*,*m*_ and *D*^*WRITE*^_*t*,*m*_ are the sets of data used by *t* that exist on *m* and that are accessed in *read* mode and *write* mode, respectively.

We define the sum of all the pieces of data hosted (*LS_SDH*) with the score given by

(6) $$LS\_SDH(m,t) = \sum\nolimits_{d \in {D_{t,m}}} d.{\rm{size}}.$$

The core idea of *LS*_*SDH* is to consider that the memory node that already hosts the largest amount of data (in volume) needed by *t* is the one where *t* has to be executed.

If all the tasks use different/independent pieces of data and each of them is used once, then we except that both *MC_StarPU* and *LS*_*SDH*(*m*,*t*) return meaningful scores. However, there are other aspects to consider. For example, if there is a piece of data duplicated on every node it should be ignored. Moreover, we can also consider that a piece of data used in *read* is less critical than the ones used in *write* for multiple reasons. A piece of data used in *read* might be used by several tasks (in *read*) at the same time, and thus the transfer cost only impacts the first task to be executed on the memory node. In addition, a piece of data in *write* is expected to be used in *read* later on, which means that moving a piece of data that will be accessed in *write* on a memory node, partially guarantees that this data will be re-used soon. Finally, writing on a set of data invalidates all copies on other memory nodes. Thus, we define three different formulas based on these principles, where we attribute more weight to the *write* accesses to reduce the importance of the *read* accesses.

The LS_SDH^2^ is the score given by summing the amount of data already on a node, but the difference with *LS*_*SDH* is that each data in *write* is counted in a quadratic manner

(7) $$LS\_SD{H^2}(m,t) = \left({\sum\limits_{d \in D_{t,m}^{READ}} d.{\rm{size}}} \right) + \left({\sum\limits_{d \in D_{t,m}^{WRITE}} d.{\rm{siz}}{{\rm{e}}^2}} \right).$$

Alternatively, we propose the *LS*_*SDHB* score where we sum the amount of data on a node but we balance the data in *write* with a coefficient θ. Moreover, we consider that for the same amount of data on two memory nodes, the one that has more pieces of data should be prioritized. In other words, transferring the same amount of data but with more items is considered more expensive. The formula is given by

(8) $$LS\_SDHB(m,t) = \left({\sum\limits_{d \in D_{t,m}^{READ}} d.{\rm{size}}} \right) + \left({{\rm{\theta }} \times \Omega (D_{t,m}^{WRITE}) \times \sum\limits_{d \in D_{t,m}^{WRITE}} d.{\rm{size}}} \right).$$

We set θ = 1,000 for the rest of the study as it provides an important load to the data in *write* without canceling the cost of huge transfer for data in *read*.

Finally, we propose the *LC*_*SMWB* cost formula

(9) $$\eqalign{LC\_SMWB(m,t) = \left({\sum\limits_{d \in D_{t,\neg m}^{READ}} d.size} \right) \cr+ \left({\sum\limits_{d \in D_{t,\neg m}^{WRITE}} d.size \times 2 \times {{\Omega (t.data \cap WRITE)} \over {\Omega (t.data)}}} \right).}$$

In *LC*_*SMWB*, we sum the amount of data that is going to be moved, but we use an extra coefficient for the data in *write*. This coefficient takes the value 1 if all the data used by *t* are in *write*, but it gets closer to 2 as the number of data dependencies in *read* gets larger than the number of data dependencies in *write*.

### Examples of memory node selection

Table 1 illustrates how the formulas behave and which memory nodes are selected for different configurations. This example shows that the formulas can select different memory nodes depending both on the number of data dependencies in *read*/*write* and their sizes.

**Table 1:** Examples of memory node selection by the proposed DLAF for different tasks and data configurations.

Tasks(Data/access mode/size, …)	MN0 hosts	MN1 hosts	MN2 hosts	*LS_DH* winner	*LS*_*SDH*^2^ winner	*LS*_*SDHB* winner	*LC*_*SMWB* winner
T(A/R/1, B/W/1)	A	A	B	MN{0,1,2}	MN{0,1,2}	MN2	MN2
T(A/R/1, B/W/1)	A	A B	B	MN1	MN1	MN1	MN1
T(A/W/1, B/W/1, C/W/2)	A B	C	A C	MN2	MN2	MN2	MN2
T(A/W/1, B/W/1, C/W/1)	A B	A B	A C	MN{0,1,2}	MN{0,1,2}	MN{0,1,2}	MN{0,1,2}
T(A/R/2, B/R/1, C/W/2, D/W/2)	A B	A C	C D	MN2	MN2	MN2	MN2
T(A/W/10, B/W/11, C/W/18, D/W/11)	A D	C	B D	MN2	MN1	MN2	MN2
T(A/W/10, B/W/11, C/W/22, D/W/11)	A D	C	B D	MN{1,2}	MN1	MN2	MN{1,2}

**Note:**

The memory nodes are labeled MN and in the case the scores assign the best values to more than one memory nodes, all of them are written inside brackets.

### Automatic DLAF selection

**Figure 4:**
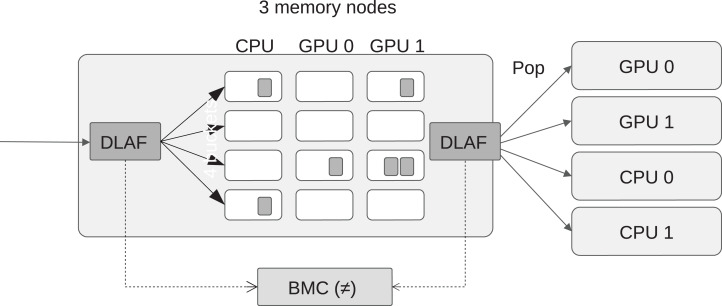
View of the best memory node difference (BMD), which is computed by counting the number of difference returned by the DLAF between the moment when a task is pushed or popped.

We propose several data locality affinity formulas (DLAF) but only one of them is used to find out the best memory node when a newly ready task is pushed into the scheduler. We describe here our mechanism to automatically select a DLAF during the execution by comparing their best memory node difference (BMD) values. A BMD value indicates the robustness of a DLAF by counting how many times it returns a different node id when a task is pushed or popped. More precisely, every time a task *t* is pushed, we call a DLAF to know which of the memory nodes is selected to execute the task, and we store this information inside the scheduler. Then, every time a task is popped, we call again the same DLAF to know which of the memory node seems the more appropriate to execute the task, and we compare this value with the one obtained at push time, as illustrated by Fig. 4. If both values are different, we increase the BMD counter. A low BMD value means that the DLAF is robust to the changes in the memory during the push/pop elapsed time. We consider that this robustness is a good metric to automatically select a DLAF, and thus we continually compared the BMD counters of all DLAF, and use the one that has the lowest value to select the list where to push the tasks.

### Iterating order on the lists of the grid (pop)

In this section, we describe how the workers iterate over the task-lists of *G*.

#### Distance between memory nodes

First, we build a distance matrix between the memory nodes. We defined the data transfer speed between memory nodes as an inverse of the distance; the distance is given by StarPU and it is the time that takes to move a piece of data from one memory node to another

(10) $${\rm{distanc}}{{\rm{e}}_{{\rm{transfer}}}}(i,j) = {\rm{normalize}}(starpu\_transfer\_predict(j,i{,1024^3})).$$

However, it is important to remember that our scheduler is based on priorities and thus we also use a second metric to look at the difference in terms of priorities between the workers of different memory nodes. More precisely, we define a priority distance between workers of different memory nodes by

(11) $${\rm{distanc}}{{\rm{e}}_{{\rm{priority}}}}(i,j) = 1-{{\sum\nolimits_{k = 1}^B |P(i,k)-P(j,k)|} \over {({\rm{max}}(N{P_i},N{P_j}) + 1) \times ({\rm{max}}(N{P_i},N{P_j}) + 2)/2}}.$$

The numerator of the fraction provides a difference factor between *i* and *j*, whereas the denominator part ensures that the values stays between 0 and 1. The value 0 is obtained when two workers used the same priority indexes. They access the same buckets in the same order. In Table 2, we provide examples of the priority distance for two array indexes.

**Table 2:** Priority distance examples between buckets/priorities indexes of *i* and *j*.

Priorities for *i*	Priorities for *j*	distance_priority_ (*i*,*j*)
1 2	2 1 0	1−0.4
1 2	0 1	1−0.2
1 2	0 1 2	1−0
3 1 2	0 1 2 3	1−0.26
3 1 2	0 1 3 2	1−0.26
3 1 2	0 3 2 1	1−0.13

Finally, we use both distance coefficients to find a balance between priorities and memory transfer capacities, and we obtain the final measure with

(12) $${\rm{distance}}(i,j) = \left({{\rm{distanc}}{{\rm{e}}_{{\rm{priority}}}}(i,j) \times {\rm{\alpha }}} \right) + \left({{\rm{distanc}}{{\rm{e}}_{{\rm{transfer}}}}(i,j) \times (1-{\rm{\alpha }})} \right).$$

From Eq. (12), two memory nodes are close if they are well-connected and if their priorities (how their workers iterate on the buckets) are different.

#### Prioritizing locality/priorities in the access orders

Using the distance matrix between the memory nodes, two straightforward access orders can be considered. In the first one, we consider that data locality is more critical than the priority of the tasks; In this case, a worker iterates on all the lists related to its memory node following the priority order, and only if it cannot find a ready task it looks at the lists of the second closest memory node. The workers iterate over *G*(*b*,*m*) with an outer loop of indexes *m* and an inner loop of index *b* (column-by-column). In a second case, we chose priority over data locality; In this case, a worker iterates with an outer loop of indexes *b* and an inner loop of index *m* (row-by-row). One drawback of the locality-oriented access is that it pushes the priorities in the background, which means that a local task of low priority should always be done before a less local task of higher priority. On the other hand, the priority oriented access breaks the locality benefit because a worker looks at all the memory nodes’ task-lists one priority after the other. Hence, both approaches are balanced using subgroups in this study.

#### Memory node subgroups

We propose that each memory node sees the others as two separate groups. The idea is to maximize the exchanges with the first group of size *S*, and use the second group only to steal tasks to avoid being idle. To do so, we use a locality coefficient *l* that correspond to the number of consecutive buckets that are queried before going to the next memory node. The iterations on the grid *G* are done so that the worker looks at the *l* first buckets of its memory node, then at the *l* first buckets of its *S* closest memory nodes. This is done until all buckets of the worker’s memory node and the *S* subgroups have been scanned. Then, in a second stage, the other memory nodes, from *S* + 1 to *M*, are scanned bucket after bucket. Both *S* and *l* parameters can be different for each memory nodes.

An example of this access order strategy can be seen in Table 3. With the settings given in the example, we use *l* = 2 for the CPU workers, see Table 3B. Consequently, the CPU workers look at two buckets of the CPU memory node lists, before looking at the GPU lists.

**Table 3:** Access list examples for a configuration with one CPU and two GPUs (three memory nodes in total).

(A) Distance matrix from Eq. (12).			
	CPU	GPU-0	GPU-1
CPU	0	0.5	1
GPU-0	0.5	0	1
GPU-1	0.5	1	0

(B) Access order for CPU workers.				
Priorities	Buckets	G(*,CPU)	G(*,GPU-0)	G(*,GPU-1)
3	G(3,*)	7	11	10
2	G(2,*)	6	9	8
1	G(1,*)	1	5	4
0	G(0,*)	0	3	2

(C) Access order for GPU-0 workers.				
Priorities	Buckets	G(*,CPU)	G(*,GPU-0)	G(*,GPU-1)
	G(1,*)	5	4	8
	G(2,*)	3	1	7
	G(3,*)	2	0	6

(D) Access order for GPU-1 workers.				
Priorities	Buckets	G(*,CPU)	G(*,GPU-0)	G(*,GPU-1)
	G(1,*)	5	8	4
	G(2,*)	3	7	1
	G(3,*)	2	6	0

**Note:**

We use four buckets, but the tasks of bucket zero are only active on CPU. The priorities—the order of access to the buckets—is reversed for the GPU workers. *S*, the size of closed memory node subgroup, is set to two for the CPU and to one for the GPUs. Finally, the locality factor *l* is two for both.

## Performance Study

### Configuration

The following software configuration was used: GNU compiler 6.2, CUDA Tookit 9.0, Intel MKL 2019 and StarPU^1^1We created our scheduler on the master branch of the official repository https://scm.gforge.inria.fr/anonscm/git/starpu/starpu.git at commit id 22e8e132e0e6c09c9a5d4539d46b3d59503749e7. We set the environment variables *STARPU_CUDA_PIPELINE=4*, *STARPU_PREFETCH=1* and *STARPU_DISABLE_PINNING=0*. From Eq. (12), we defined α = 0.5, and as a result the closest memory node to any GPU was always the CPU. StarPU supports multi-streaming capability of modern GPUs by running multiple CPU threads to compute on the same GPU. This is controlled by *STARPU_NWORKER_PER_CUDA* and we used different values depending on the hardware and the application that was run. The set values were application specific. The automatic DLAF selection, described in the section “Automatic DLAF Selection,” was based on *LS*_*SDH*, *LS*_*SDH*^2^, *LS*_*SDHB* and *LC*_*SMWB*, but excluded LaRU and *MC_StarPU*.

### Hardware

We used two different configurations and we refer to each of them using their corresponding GPU model.

- **P100** Is composed of 2 × Dodeca-core Haswell Intel Xeon E5-2683 v4 2,10 GHz, and 2 × P100 GPU (DP 4.7 TeraFLOPS).
- **K40** Is composed of 2 × Dodeca-core Haswell Intel Xeon E5-2680 v3 2,50 GHz and 4 × K40 GPU (DP 1.43 TeraFLOPS).

### Applications

We studied three applications to assess our method. Two of them were linear algebra applications that already used StarPU and Heteroprio. Hence, no further development was needed inside them since the interfaces of Heteroprio and LAHeteroprio are similar. The third one was a stencil application that we modified to be able to use Heteroprio/LAHeteroprio.

- **QrMumps** This application uses four different types of tasks and three of them can be run on the GPUs. We used *STARPU_NWORKER_PER_CUDA=16* on P100, and *STARPU_NWORKER_PER_CUDA=7* on K40. The test case was the factorization of the TF18 matrix^2^2The matrix had been taken from the SuiteSparse Matrix Collection at https://sparse.tamu.edu/.
- **SpLDLT** This application uses four different types of tasks and only one of them can run on the GPUs. Consequently, to select a task for a GPU, there is no choice in terms of bucket/priority but only in terms of memory node. We used *STARPU_NWORKER_PER_CUDA=18* on P100, and *STARPU_NWORKER_PER_CUDA=11* on K40. The test case was the Cholesky factorization of a 20,000 × 20,000 matrix.
- **StarPU-Stencil** This application is a stencil simulation of the game life, which is available as an example in the StarPU repository. It uses only one type of tasks that can run on CPU or GPU. Consequently, to select a task for any of the processing unit, there is no choice in terms of bucket/priority but only in terms of memory node. We used *STARPU_NWORKER_PER_CUDA=3* on P100 and K40. The test case was a grid of dimension 1,024^3^ executed for 32 iterations.

### Metrics

In our tests, we evaluated two different speedups. The first was the *speedup-from-average* (SFA), which represents the average execution times of Heteroprio based for six executions, divided by the average execution times of a target for six executions. The second was the *speedup-from-minimum* (SFM), which represents the lowest execution time of Heteroprio divided by the lowest execution time of a target, therefore, both were obtained from a single execution. The SFA provides information of the average performance that can be expected whereas the SFM provides information about the variability and gives us an idea of what could be achieved if the executions were always perfect.

### Evaluation of the locality coefficient for all DLAF

**Figure 5:**
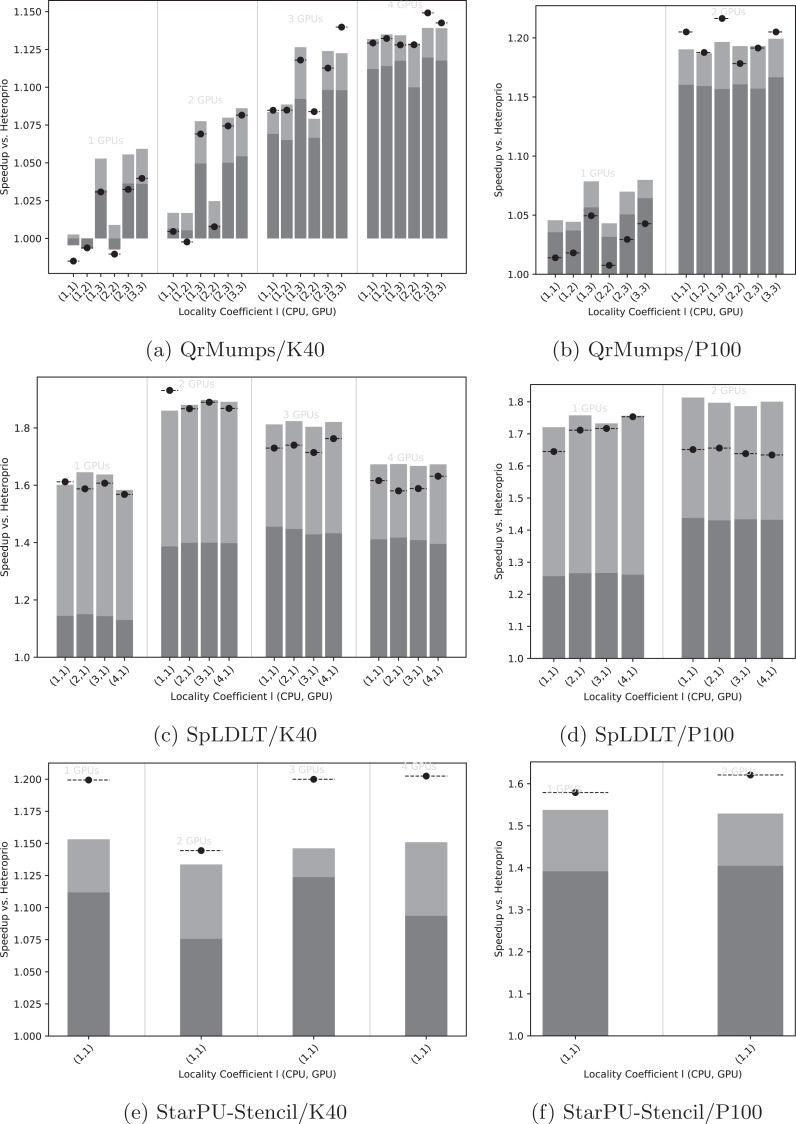
Speedup results of LAHeteroprio against Heteroprio for QrMumps (A, B), SpLDLT (C, D) and StarPU-Stencil (E, F) on K40 or P100 configurations. The *x*-axis is used of the different *l* pairs of the form (*l*_CPU_, *l*_GPU_). The gray bars (*▪*) represent SFA for all DLAF and gives an idea of the speedup of LAHeteroprio, here each configuration is executed six times. The light gray bars (*▪*) represent the SFM of the DLAF with the best speedup in average. The lines (− • −) represent the SFM using the best execution times among all DLAF, that is the speedup when we compare the best single execution using Heteroprio and LAHeteroprio.

We first evaluated the effect of the locality coefficient *l*, described in the section “Memory node subgroups,” on the execution time and summarized the results in Fig. 5. Then, we looked at the speedup of LAHeteroprio against Heteroprio for different *l* settings with three different comparisons. In the first one, we used all the average execution times obtained using LAHeteroprio without dissociating the different DLAF; in the second one we computed the speedup using only the best DLAF (with the lowest average), and in the third one we compared the unique best execution over all of both Heteroprio and LAHeteroprio.

Focusing on QrMumps, it can be seen in Figs. 5A and 5B that the best performance was obtained when we prioritized the locality for the GPU with *l*_GPU_ = 3. The locality coefficient for the CPU seems less critical and the speedup is more or less the same for all *l*_CPU_ values. When the number of GPUs increases, the influence of *l* decreases, and we had similar executions with two P100 GPUs or four K40 GPUs for all *l* values. However, the speedup against Heteroprio was still significant, which means that splitting the buckets into several lists is beneficial as soon as the workers pick first in the list that corresponds to their memory node for their highest priority bucket. Also, it seems that the way they iterate on the grid does not have any effect.

The results for SpLDLT are provided in Figs. 5C and 5D. Here, the impact of *l* seems to be limited, but it is worth remembering that the GPU can only compute one type of task. On the other hand, the speedup obtained using all DLAF was unstable and significantly lower compared to the speedups obtained when we used only the best DLAF. This suggests that there are significant differences in performance among the different DLAF and also that some of them are certainly not efficient. The results that we provide in the next section corroborates this hypothesis.

The results for StarPU-Stencil are provided in Figs. 5E and 5F. There is no choice in the value *l* because there is only one type of task. The speedup obtained using all DLAF was unstable and significantly lower compared to the speedups obtained when we used only the best DLAF, which again suggests that the different DLAF provide heterogeneous efficiency.

### Execution details

**Figure 6:**
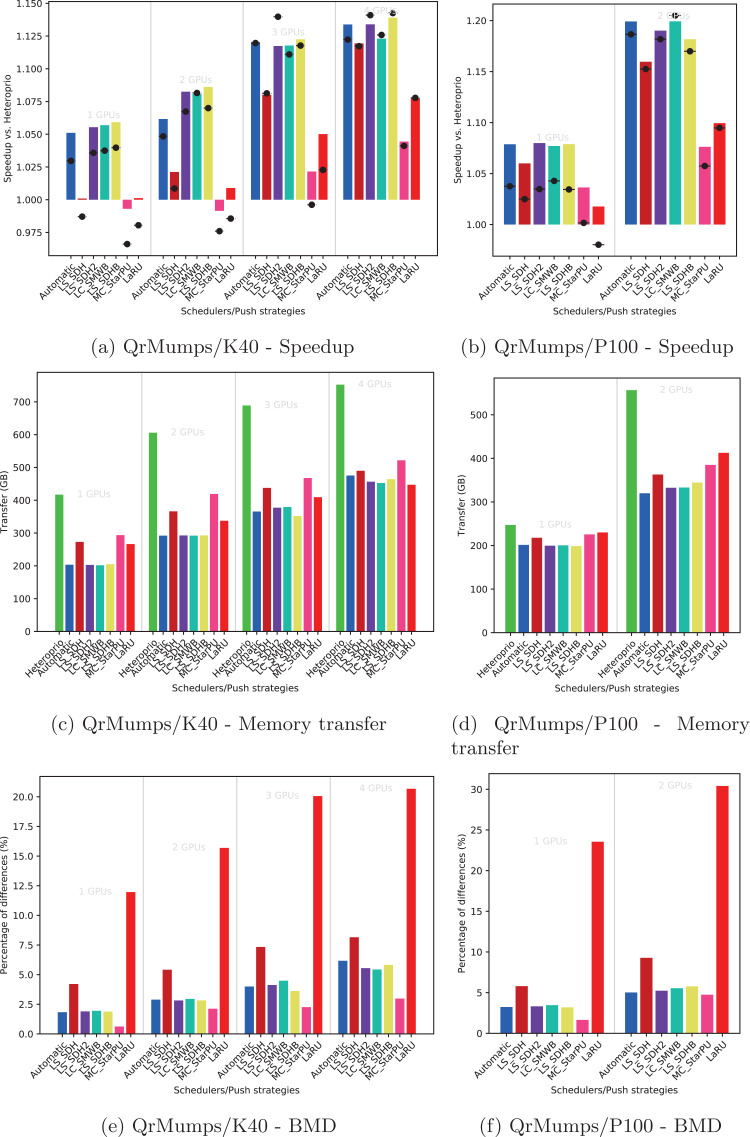
Execution details for QrMumps on K40 or P100 configurations for a locality coefficient *l* = (3, 3). The speedup (A, B) includes SFA (▪) and SFM (− • −). The memory transfers (C, D) and BMD (E, F) are average values.

**Figure 7:**
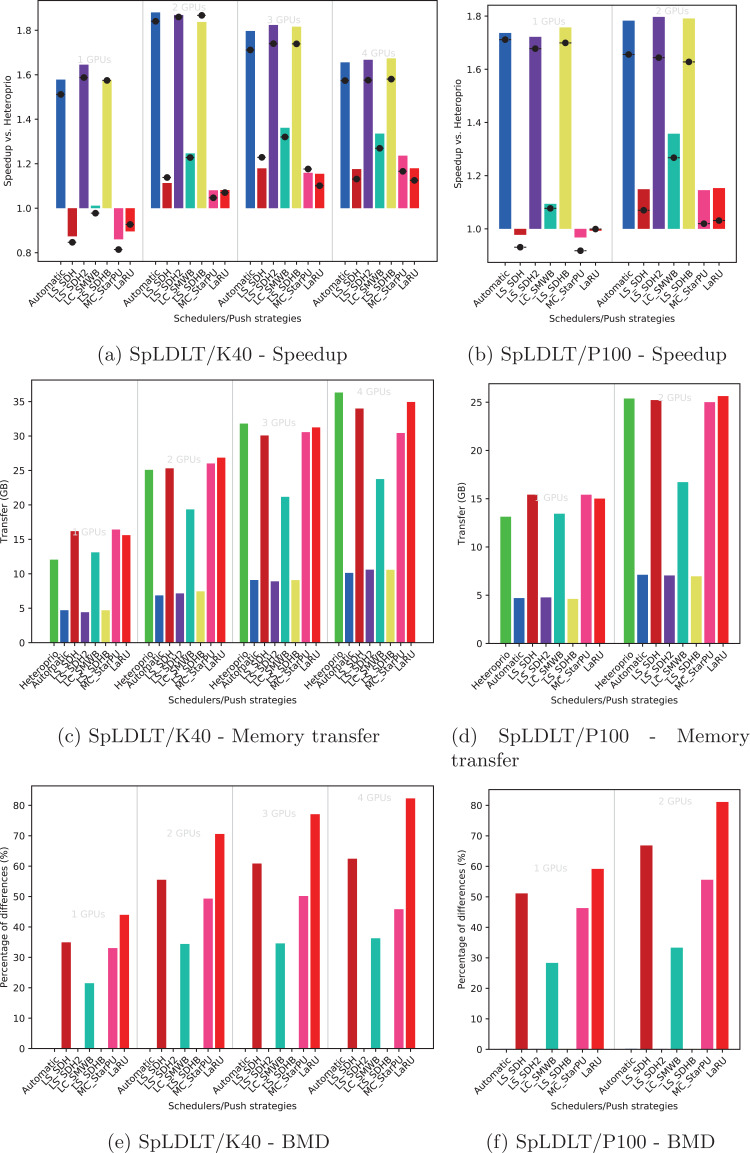
Execution details for SpLDLT on K40 or P100 configurations for a locality coefficient *l* = (2, 1). The speedup (A, B) includes SFA (▪) and SFM (− • −). The memory transfers (C, D) and BMD (E, F) are average values.

**Figure 8:**
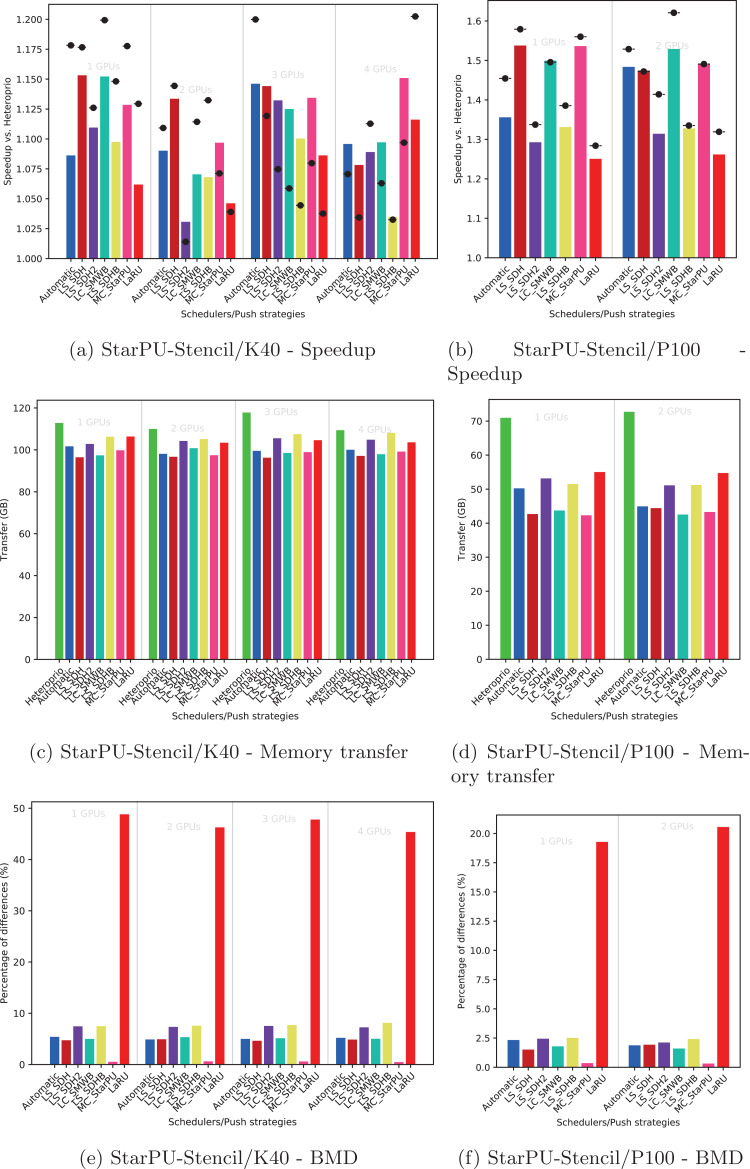
Execution details for StarPU-Stencil on K40 or P100 configurations for a locality coefficient *l* = (2, 1). The speedup (A, B) includes SFA (▪) and SFM (− • −). The memory transfers (C, D) and BMD (E, F) are average values.

Using the performance results of section “Evaluation of the Locality Coefficient for all DLAF,” we used a *l* = (1,3) for QrMumps, and a *l* = (3,1) for SpLDLT. We evaluated the performance of the different DLAF described in the section “Task Insertion in the Grid with Locality Evaluation (push),” looking for the speedup against Heteroprio, the amount of memory transfer, and the BMD, see Figs. 6–8.

#### Speedup

We provide the speedup obtained with our method against Heteroprio in Figs. 6A and 6B for QrMumps, Figs. 7A and 7B for SpLDLT, and Figs. 8A and 8B for StarPU-Stencil. For all configurations, the *LaRU* and *MC*_*StarPU* formulas did not significantly improve the execution, furthermore, they were slower than Heteroprio in some cases. For *LaRU*, this means that having one piece of data already on the memory node and neglecting the others is not efficient. Meanwhile, for *MC*_*StarPU*, it means that putting a task on the memory node for which it is the cheapest in terms of data transfer is not the best choice. This is not surprising, since this kind of decision would make sense if we have only one task to compute. However, we clearly see that in the present study, when we had to deal with a graph of tasks, where the data were used concurrently and could be re-used by other tasks, this was not accurate. Nevertheless, this result could also have been affected from inaccurate predictions made by StarPU.

Comparing the different DLAF, it can be seen that both *LS*_*SDH*^2^ and *LS*_*SDHB* significantly improved the three applications. *LC*_*SMWB* was competitive for QrMumps and StarPU-Stencil but not for SpLDLT, and *LS*_*SDH* was competitive for StarPU-Stencil but not for QrMumps and it had poor performance for SpLDLT. The main difference between *LS*_*SDH*^2^/*LS*_*SDHB* and *LC*_*SMWB*/*LS*_*SDH* is that the second ones are not giving an important load to the pieces of data used in *write*, and *LS*_*SDH* does not even make a distinction between *read* and *write*. It seems that taking into account *write* is important for QrMumps and SpLDLT but not for StarPU-stencil. On the two linear algebra applications, the tasks transform the blocks of the matrix, and many of the blocks are written several times before being read multiple times. On the contrary in StarPU-stencil, each block is written once per iteration and read only to compute the close neighbors.

While the results from the different DLAF are diverse, our automatic formula selection, described in the section “Automatic DLAF selection,” was efficient and always close to the best execution. Consequently, there is no need to try the different DLAF as the automatic selection is reliable.

#### Transfer

The total amount of memory transfer obtained with our method and Heteroprio are provided in Figs. 6C and 6D for QrMumps, Figs. 7C and 7D for SpLDLT, and Figs. 8C and 8D for StarPU-Stencil.

For QrMumps, all approaches used in this study reduced the total memory transfer. However, a decrease of the memory transfer does not necessary mean having better performance. For example, for the K40 configuration, and with either one or two GPUs, *MC*_*StarPU* drastically reduced the amount of data transfer compared to Heteroprio, see Fig. 6C, but it had a negative speedup, see Fig. 6A. It means that, even if in all LAHeteroprio-based executions the workers iterated similarly on *G*, the placement of the tasks on the grid can be quite efficient in terms of transfer, but it penalized the whole execution.

In the case of SpLDLT, the memory transfer did not decrease compared to Heteroprio when *MC*_*StarPU*, *LaRU*, or *LS*_*SDH* were used. This further supports our idea that the data in *write* should count more than the data in *read*. Moreover, *LC*_*SMWB* balances the data in *write* but only with a factor 2 at most; even if it reduced the memory transfer compared to Heteroprio, the reduction was not as large compared with *LS*_*SDH*^2^/*LS*_*SDHB*. Finally, when we used SpLDLT the amount of memory transfer and the execution time were reduced.

Looking at the results of StarPU-Stencil, the memory transfer reduction was not as strong as for QrMumps. In addition, there is a correlation between the transfer reduction and the resulting speedup, such that the lowest amount of transfer were obtained with *LS*_*SDH*, *LS*_*SMWB* and *LS*_*SDHB* for most of the configurations.

Again, the automatic mode is efficient and even when one of the DLAF is not competitive, for instance *LC*_*SMWB* in the case of QrMumps/SpLDLT or *LC*_*SDH*^2^ for StarPU-Stencil, the automatic system is robust enough to make correct decisions and remains competitive.

#### BMD

We provide the BMD values for the different DLAF in Figs. 6E and 6F for QrMumps, Figs. 7E and 7F for SpLDLT, and Figs. 8E and 8F for StarPU-Stencil.

For QrMumps, the BMD values were low for all formulas except *LS*_*SDH* and *LaRU*. These measures proof that *LS*_*SDH* is sensitive to the data changes that happen in the time that takes a pushed task to be popped. Furthermore, this is due to its formula as it considers the data in *read* or *write* to be the same. On the other hand, *MC*_*StarPU* was stable with a small BMD value. However, this is surprising, because the high value for *LS*_*SDH* illustrates the volatility of the data, and thus *MC*_*StarPU* should also be sensitive to the changes that happened between push/pop.

For SpLDLT and StarPU-Stencil, we observed a clear relation between the BMD values and the speedup. The formulas that did not provide a speedup are the ones with the highest BMD values. This validates the construction of our automatic method that uses the DLAF with the lowest BDM.

In the three applications, the *LaRU* has a special meaning when looking at the BMD value. When a task is pushed, *LaRU* returns the id of the memory node of the worker that push the task and similarly, when a task is popped, *LaRU* returns the id of the memory node of the worker that pop the task. Therefore, the *LaRU’*s BDM value is the percentage of tasks that are pushed and popped by worker related to different memory nodes. Therefore, we see that in QrMumps up to 30% of the tasks were stolen but this number grow up to 50% for StarPU-Stencil and 80% for SpLDLT.

#### Summary of the evaluation

The speedup obtained with LAHeteroprio was really significant. In most cases, there was a proportional relation between memory transfer and execution time, which means that reducing memory transfer caused a reduction in the time needed to execute the task. The BMD metric is valuable to evaluate the robustness of DLAF and it can be used to predict its performance. Moreover, our automatic DLAF selection based on BMD was highly competitive with a speedup close to the best-achieved executions. Finally, LAHeteroprio reduced the amount of memory transfer with any number of GPUs for the three applications.

## Conclusion

We have improved our Heteroprio scheduler with a new mechanism that considers data locality. The new system divides the task buckets into as many lists as there are memory nodes. We have created different formulas to evaluate the locality of a task regarding a memory node, and we found that formulas that omit many parameters (as the use of the StarPU prediction functions) provide a low performance; this is probably due to the neglect of the type of accesses of the tasks on the data. Nevertheless, we have shown that locality evaluation is more sensitive to *write* accesses and this has been validated with the results of the BMD metric. Concerning the pop strategy, it is necessary to set the locality coefficient to the largest value for the GPUs, to ensure that workers focus on locality before priorities. It is possible to use our new scheduler, without introducing additional information or modification, using our automatic DLAF selection system, which is close to the best executions in most cases. Finally, our new scheduler improves the performance of QrMumps, SpLDLT and StarPU-Stencil by 30%, 80% and 30%, respectively. It also reduces the data transfer more than 50%.

In terms of perspective, the scheduler could still be improved on different aspects. It could be beneficial to change the distance between the memory nodes at runtime, which means changing the victims of the work stealing and even having workers of the same memory node that steal the tasks on other memory nodes. In addition, the original priorities of the scheduler are set per architecture, and the new locality heuristic is set per memory node, but a finer approach could be interesting even if it has a challenging tuning and setup. For example, we could have one worker per GPU that uses a different access order over the buckets with the objective of avoiding some transfers. Finally, we would like to study LAHeteroprio on other kinds of applications with more diverse types of tasks, and on different type of hardware configurations.

## Supplemental Information

10.7717/peerj-cs.190/supp-1 Supplemental Information 1 Source code of the LAHeteroprio scheduler. This file is the scheduler used to generate the results of the paper. It must be plugged into StarPU.

10.7717/peerj-cs.190/supp-2 Supplemental Information 2 SpLDLT results for the different configurations.

10.7717/peerj-cs.190/supp-3 Supplemental Information 3 Stencil results for the different configurations.

10.7717/peerj-cs.190/supp-4 Supplemental Information 4 QrMumps results for the different configurations.

## Additional Information and Declarations

### Data Availability

The following information was supplied regarding data availability: The Supplemental Files include the source code of the scheduler and the details of the executions of QrMumps and SpLDLT in two raw data/CSV files. These results were used to generate the figures for the article but they also contain additional information not presented in the article.
